# Two types of critical cell density for mechanical elimination of abnormal cell clusters from epithelial tissue

**DOI:** 10.1371/journal.pcbi.1010178

**Published:** 2022-06-13

**Authors:** Sang-Woo Lee, Yoshihiro Morishita

**Affiliations:** Laboratory for Developmental Morphogeometry, RIKEN Center for Biosystems Dynamics Research, Kobe, Japan; Oxford, UNITED KINGDOM

## Abstract

Recent technological advances in high-resolution imaging and artificial modulation of genetic functions at different times and regions have enabled direct observations of the formation and elimination of abnormal cell populations. A recent trend in cell competition research is the incorporation of cell mechanics. In different tissues and species, abnormal cells developing in epithelial tissues are mechanically eliminated by cell contraction via actomyosin accumulation at the interface between normal and abnormal cells. This mechanical cell elimination process has attracted attention as a potential universal defense mechanism. Here, we theoretically examined the conditions for mechanical elimination of growing abnormal cell populations. Simulations and mathematical analyses using a vertex dynamics model revealed two types of critical cell density associated with mechanical elimination of abnormal cell clusters. One is a subtype of homeostatic density, in which the frequencies of spontaneous mechanical cell elimination and proliferation are balanced, even if no explicit dependence of proliferation or apoptosis on the cell density is assumed. This density is related to the mechanical stability of a single cell. The other is density related to mechanical stability as a cell population under external pressure. Both density types are determined by tissue mechanical properties. In solid tissues, the former type is reached first as the intensity of interfacial contraction increases, and it functions as a critical density. On the other hand, the latter type becomes critical when tissues are highly fluid. The derived analytical solution explicitly reveals the dependence of critical contractile force and density on different parameters. We also found a negative correlation between the proliferation rate of abnormal cells and the likelihood of the abnormal cell population expanding by escaping elimination. This is counterintuitive because in the context of cell competition, fast-growing cell populations generally win. These findings provide new insight into, and interpretation of, the results from experimental studies.

## Author summary

High-resolution imaging techniques have revealed that abnormal cells developing in epithelial tissues are mechanically eliminated via contraction at the interface between the abnormal cells and normal surrounding cells. This phenomenon is seen in various species and tissues and thus is regarded as a primitive defense system against precancerous cells common to all animals. For comprehensive understanding of this potential defense system, we derived mathematical conditions to achieve mechanical elimination of growing abnormal cell populations. We identified two characteristic cell density types associated with successful mechanical elimination of abnormal cell clusters. Both are determined by tissue physical properties, and the smaller of the two functions as a critical density above which abnormal cell populations cannot exist. We also found a counterintuitive phenomenon in which slower proliferation of abnormal cells promotes their growth as a population. Our results will help elucidate the mechanisms of intrinsic tissue defenses against cancer from the perspective of cell mechanics.

## Introduction

Epithelial tumors begin with the emergence of a small population of abnormal cells (e.g., via mutations in tumor suppressor genes), and eventually these abnormal cells infiltrate large areas of the body via growth and increase the risk of malignancy due to further mutations. The formation of a population from a single abnormal cell is a microscopic and random event, which has rendered research of this process difficult. Advances in technologies that enable artificial induction of gene abnormalities, as well as enhanced imaging techniques, have finally made it possible to observe these processes, which have become well studied in recent years, especially from the viewpoint of cell competition [1,2].

In the field of cell competition, the study of related chemical signal transduction systems was originally the primary focus [3–11]; however, cell mechanics have recently become relevant as well [12–25]. For instance, cultured Madin–Darby canine kidney II (MDCK) cells are hypersensitive to compaction after knockdown of the polarity gene *scribble*, and their interaction with wild-type cells causes compaction of knockdown cells and elevation of p53 levels in them, resulting in cell death [16,26]. When apoptosis is density dependent, the density threshold determines the tolerant density specific to each cell population, and mixing proliferating cell populations possessing different thresholds will result in survival of the population with the highest threshold. This phenomenon can be explained by homeostatic cell density/pressure, a physics concept proposed by Basan et al. [27]. In addition, misspecification of a subset of cells in the wing imaginal disc of Drosophila can trigger actomyosin accumulation at the borders between normal and abnormal cells, leading to contraction at the cell interface [14,28]. When the population size of abnormal cells is small, the cells are eliminated by cell death via this contraction, and when the population size is intermediate, a cyst is created [14]. The contraction of apoptotic cells with an internal ring-like structure composed of F-actin has also been observed [29,30]. Furthermore, in the context of organ development, increased density causes live-cell extrusion, without apoptosis, from the apical or basal side of an epithelial sheet [19,20]. In this manner, mechanical elimination processes such as mechanically driven apoptosis and live-cell extrusion have been observed in many different species and tissues and have attracted attention as a potential mechanism of homeostasis or protection against cancer.

Mechanical elimination of abnormal cells has been investigated both experimentally and theoretically. While experimental research focuses on the examination of actual specific cells or tissues [19], one of the advantages of a theoretical approach is that it allows comprehensive examination of elimination conditions under various situations and understanding of the overall perspective of the elimination process. In our previous study, motivated by the above findings, using numerical simulations of a vertex dynamics model and mathematical analyses, we derived the general conditions for mechanically eliminating a single abnormal cell via contraction of the cell edge. The minimum force required for elimination (termed critical contractility) was given as a function of different quantities such as the mechanical and geometrical cell parameters on a targeted abnormal cell [31].

In contrast, in more realistic situations, abnormal cells continue to proliferate to form a population. Thus, in the present study, we numerically and analytically examined the conditions necessary for elimination of growing abnormal cell populations via contraction at the interface between normal and abnormal cells. Our analysis revealed two types of critical density in abnormal cell populations for achieving mechanical elimination of the populations. The first is a subtype of the homeostatic density previously introduced by Basan et al. [27] and is related to the mechanical stability of a single cell. The other is related to the mechanical stability as a cell population. The values of both densities are determined by tissue physical properties (e.g., bulk and shear moduli), and the smaller of the two is the critical density above which abnormal cell clusters are completely eliminated. In addition, the analytical solution we derived also provides a good estimate of the critical interfacial contractility required for eliminating abnormal cell clusters with different physical properties.

## Results

### A vertex dynamics model for epithelial tissue growth simulation

**Fig 1:**
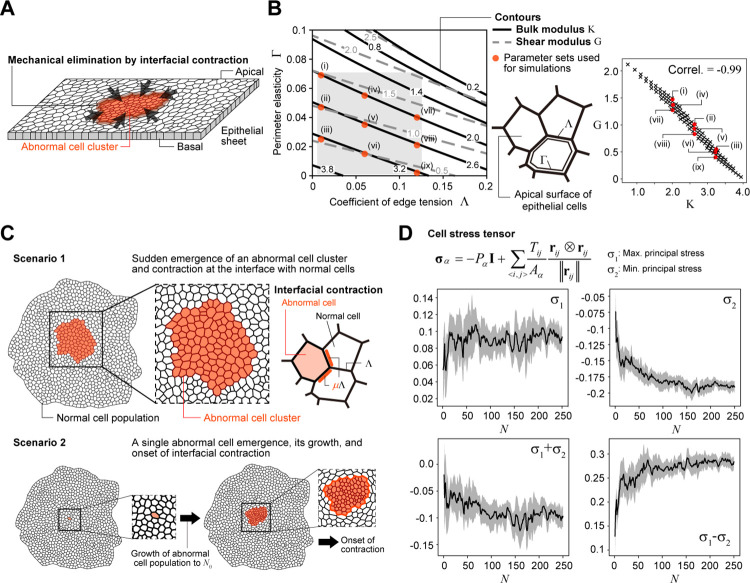
Schematic diagrams for vertex dynamics model and simulation settings. (A) A scheme for mechanical elimination of abnormal cell clusters by interfacial contraction in epithelial tissues. (B) (left) Contours of bulk modulus *K* (black lines) and shear modulus *G* (gray broken lines) in cell mechanical parameter (Λ-Γ) space and (middle) a scheme for the apical dynamics of epithelial cells with Λ and Γ. For smaller Λ and Γ values, the tissue fluidity is higher. (right) The relationship between *K* and *G*, where the values calculated at 100 points in the gray region of (Λ-Γ) space are plotted. Red closed circles in the left and right panels (labeled as (i)–(ix)) show the parameter sets used for the simulations. (C) Two types of scenarios for the simulations. Scenario 1 is a artificial/simplified situation, in which an abnormal cell cluster appears at a certain moment within a normal cell population, and immediately after its appearance, the interfacial contraction *μ*Λ comes into play (top). Scenario 2 is a more realistic situation, in which a single abnormal cell appears in a large population of normal cells and grows via cell division. Once the number of abnormal cells reach a certain size (*N*_*θ*_), interfacial contraction *μ*Λ occurs (bottom). See the text for details. (D) The relationship between the average stress state of the normal cells at the interface and the number of abnormal cells *N* (black lines). The gray regions show the standard deviation. σ_1_ and σ_2_ are the maximum (tensile, tangential to the interface) and minimum (compressive, perpendicular to the interface) principal stresses, respectively. In particular, σ_2_ and σ_1_ − σ_2_ show a clear dependence on *N*. See [37] for the calculation of cell stress tensor.

As stated in the Introduction, the mechanical elimination of abnormal cells has been reported under different situations. Here, we focus on the contraction force at the boundary between normal and abnormal cells as a mechanism for eliminating abnormal cell populations (Fig 1A) and examined the dependence of the elimination success rate on different factors using a 2D vertex dynamics model (see Methods), which describes the apical dynamics of epithelial cells [32–36]. Although the elimination of abnormal cells from the epithelium is a 3D process, here we focused on the conditions for elimination at the apical surface because shrinkage of the apical surface is initially observed in many cases of mechanical cell elimination [29].

In a vertex dynamics model, the physical properties of a cell population or tissue are determined by the coefficients of line tension (Λ) and perimeter elasticity (Γ); more quantitatively, the bulk (*K*) and shear (*G*) moduli are given as functions of Λ and Γ (see Methods and also Fig 1B (left) for contours of *K* and *G* in (Λ-Γ) space). The values of *K* and *G* are negatively correlated in a wide range of regions in (Λ-Γ) space (Fig 1B, right). For smaller values of Λ and Γ, the tissue fluidity is higher [34,37,38]. As examples, according to the values of Λ and Γ estimated in previous works, MDCK cell monolayers are more fluid (*K* = 3.2 and *G* = 0.71) [29], whereas epithelial cells in the wing disc of Drosophila are less fluid, i.e., more solid (*K* = 2.01 and *G* = 1.27) [34]. In the following simulations and mathematical analysis, we examine the elimination conditions for tissues with different physical properties.

We considered two types of cells, normal and abnormal. Unless noted otherwise, both cell types were assumed to have the same physical parameters Λ and Γ, except at the interface between normal and abnormal cells. Following our previous study on the mechanisms of mechanical elimination of a single abnormal cell [31], edge contraction was reflected by Λ; the coefficient between normal and abnormal cells was set as *μ*-fold that between normal cells or between abnormal cells, where *μ* ≥ 1 (Fig 1C).

It was assumed that the two cell types exhibit different cell cycle durations (*τ*_N_ and *τ*_A_, respectively), with the cell cycle being shorter in an abnormal cell (*τ*_A_) than a normal cell (*τ*_N_) (see Methods regarding cell cycle modeling). Unless otherwise stated, the cell cycle of normal cells was set at infinity (i.e., the situation in which only abnormal cells proliferate was considered). Since abnormal cells generally continue to grow due to dysregulated proliferation, we also assumed that *τ*_A_ is independent of abnormal cell density [39–41]. Note that we included some randomness in the cell cycle time to avoid synchronous cell division [37].

We considered two scenarios for the simulations. Scenario 1 is a somewhat artificial and simplified situation in which an abnormal cell cluster with *N*_*θ*_ cells arises at a certain moment within a normal cell population, and immediately after its appearance, the interfacial contractile force comes into play; i.e., the coefficients of the interfacial edges were set to *μ*Λ (Fig 1C, top). The abnormal cell clusters were positioned to form a circle around the center of the normal tissue. After onset of the contractile force, the abnormal cells begin to divide. The reason for evaluating the dynamics under this scenario is that, as we will see later, the densities (or average sizes) of normal and abnormal cells at the onset of the contractile force are the same, which simplifies the assumptions made when deriving the analytical solution for the elimination condition. Importantly, the basic logic behind the elimination dynamics examined in this simpler scenario, also holds in the more realistic Scenario 2 described next. Scenario 2 is a more realistic situation in which a single abnormal cell randomly arises within a large population of normal cells (e.g., ~1,000) and multiplies via cell division. Once the number of abnormal cells reaches *N*_*θ*_, contractile forces occur at the interface between the abnormal cells and surrounding normal cells (Fig 1C, bottom). This is under the assumption that surrounding normal cells can detect the appearance or existence of abnormal cells with some delay in the detection process, which should be correlated with the size of the abnormal cell population *N*_*θ*_. Thus, we dealt with *N*_*θ*_ as a parameter and examined the system dynamics for different values of *N*_*θ*_. Furthermore, as shown in Fig 1D, the stress state of normal cells at the interface shows a clear correlation with the number of abnormal cells, specifically between the minimum principal stress (compressive stress) σ_2_ and the number of abnormal cells *N*, and between the difference in principal stress σ_1_ − σ_2_ and *N*; note that σ_1_ − σ_2_ is strongly correlated with cell shape [37] (see also [37,42] for the calculation of cell stress tensor). Thus, Scenario 2 can also be interpreted as a scenario in which the surrounding normal cells recognize the existence of abnormal cell populations by the change in tissue stress, the magnitude of which is a function of *N*; in fact, myosin activity is higher at the cell edge under tissue stress during tissue development (e.g., [43–45]). Under Scenario 2, the abnormal cell density at the onset of contractility is higher than that in the surrounding normal tissue, and as will be seen, the mathematical analysis of the elimination condition is a bit more complicated. In the following sections, the simulation results under Scenario 1 are shown unless otherwise noted.

### Two types of phase diagrams for elimination success/failure

**Fig 2:**
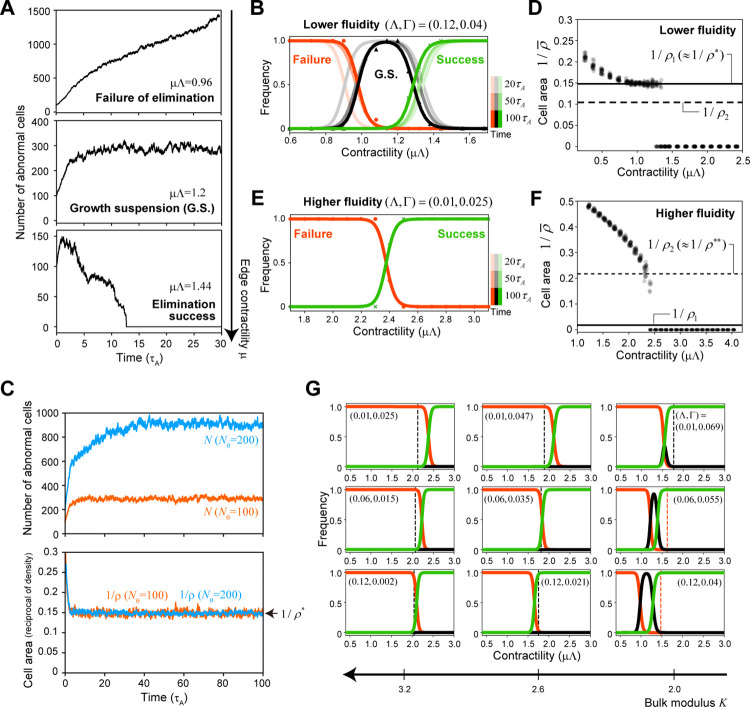
Numerical simulations showed that there are two types of phase diagrams for elimination success/failure depending on the physical property of tissues. (A) Typical time course of the number of abnormal cells in cases of elimination failure (top), growth suspension (middle), and elimination success (bottom) under lower tissue fluidity. (B, E) The dependence of elimination success on interfacial contractility *μ*Λ for tissues with lower fluidity (B) or higher fluidity (E); the frequencies of elimination failure (red), growth suspension (G.S.; black), and elimination success (green). Note that the growth suspension phase appeared when the tissue fluidity was lower. The frequencies at different time points are represented as lines with different transparencies; the curves for the different time points overlap in (E). Each point (i.e., circle, triangle, or cross) represents the average result at *t* = 100*τ*_A_ for 20 simulation runs. The thick lines were obtained by fitting the Hill functions (i.e., *f*(*x*) = *K*^*h*^/(*x*^*h*^+*K*^*h*^) for “Failure”, *g*(*x*) = *x*^*h*^/(*x*^*h*^+*K*^*h*^) for “Success”, and 1−*f*(*x*)−*g*(*x*) for “G.S.”). (C) The growth suspension phase showing that the size of the abnormal cell cluster remains nearly constant for a long period, with the size depending on the initial cluster size *N*_*θ*_ (top), but the cell density remains nearly constant independently of *N*_*θ*_ (bottom). Orange lines represent *N*_*θ*_ = 100 and blue lines *N*_*θ*_ = 200. (D, F) The time averages of the cell area ($1/\bar{\rho }$) for each simulation run in the case of *N*_*θ*_ = 100. For each *μ* value, the results from 20 simulation runs were plotted. For the tissue with lower fluidity (D), a plateau density ($\bar{\rho }$ = ρ*) appeared for intermediate values of *μ*, suggesting that ρ* is the critical density associated with mechanical elimination of abnormal cell clusters. As shown later, ρ* is almost equal to the mechanical homeostatic cell density *ρ*_1_ (see also Figs 3C and 4B). On the other hand, for the tissue with higher fluidity (F), $1/\bar{\rho }$ is an upwardly convex function at smaller *μ* values, without a plateau density, before jumping to zero at a higher value of *μ*. The cell density just before the jump, denoted as ρ**, provides another critical density for mechanical elimination different from *ρ*_1_ (see Fig 4C and the text for details). (G) The dependence of elimination success on interfacial contractility *μ*Λ for different sets of mechanical parameters; the frequencies of elimination failure (red), growth suspension (black), and elimination success (green). Each curve was obtained using the same Hill function fitting explained above. It should be noted that the phase diagrams for elimination success/failure were similar for the sets of mechanical parameters yielding the same bulk modulus value *K*(Λ,Γ). Each vertical broken line in the phase diagram shows the critical contractility *μ*_2_Λ (black) or *μ*_1_Λ (red) obtained from analytical solutions (see the text and Fig 4D). The parameter set (Λ,Γ = 0.12, 0.04) was used in (A–D) and (Λ,Γ = 0.01, 0.025) in (E and F). In (G), the nine parameter sets shown in Fig 1B were used. All results were obtained under *N*_*θ*_ = 100 and Scenario 1, except for those in (C), which were obtained under *N*_*θ*_ = 100, 200.

We performed numerical simulations for cells with different values of *K*(Λ,Γ) and found two qualitatively different elimination mechanisms, depending on the tissue physical property *K*. In the case of tissues with lower fluidity (e.g., *K* = 2), three typical time courses for the number of abnormal cells were observed with the change in relative contractility *μ* (Fig 2A and 2B): (a) for smaller values of *μ*, elimination failed, and the abnormal cell population continued growing (Fig 2A, top), (b) for sufficiently large values of *μ*, complete elimination was successful (Fig 2A, bottom), and (c) for intermediate values of *μ*, dynamic equilibrium or growth suspension was observed, in which the abnormal cell cluster maintained a certain size due to a balance between increasing cell number via proliferation and decreasing cell number via spontaneously occurring cell elimination due to mechanical instability (Fig 2A, middle). Regarding the growth suspension phase, finite time simulations cannot exactly determine if this equilibrium really exists in the limit of *t* → ∞. In this study, when the total number of abnormal cells was smaller than a certain threshold over 100 cell cycles of an abnormal cell (i.e., 100*τ*_A_), the resultant time course was classified as this phase. As shown in Fig 2B, the frequency of this phase slowly decreases with the simulation time, but in some simulations, this dynamic equilibrium state was observed to last a very long time (e.g., more than 10,000τ_A_). Biologically, suppressing the expansion of abnormal cell populations over a long period is critical for individual survival, so here we adopted the above classification with three phases, not two, i.e., elimination success/failure. Furthermore, in the growth suspension phase, the size of the abnormal cell cluster remains almost constant for a long time and its size is dependent on the initial cluster size *N*_*θ*_ at the onset of interfacial contraction (Fig 2C, top), but interestingly, the cell density of the cluster is nearly constant independent of *N*_*θ*_ (denoted by ρ*) (Fig 2C, bottom). Importantly, the value of ρ* was independent of the simulation time (S1 Fig). Plotting the average area of the abnormal cell ($1/\bar{\rho }$) against *μ* for a given *N*_*θ*_ (see Methods for the definitions of ρ* and $\bar{\rho }$) showed that $1/\bar{\rho }$ is a downwardly convex function for smaller values of *μ*. A range of *μ* values in which $\bar{\rho }$ is constant (i.e., $\bar{\rho }$ = ρ*) is observed, and $1/\bar{\rho }$ jumps to zero at a larger *μ* (Fig 2D), suggesting that the density in the growth suspension phase ρ* is a critical density associated with mechanical elimination of abnormal cell clusters.

On the other hand, when the tissue has higher fluidity (e.g., *K* = 3.2), the profile of $\bar{\rho }$ is qualitatively different from that when the tissue has lower fluidity; $1/\bar{\rho }$ is an upwardly convex function for smaller *μ* values and decreases continuously with increasing *μ* values, finally jumps to zero at a certain value of *μ* without entering the growth suspension phase (Fig 2E and 2F). We denote the cell density just before the jump as ρ**, which appears to be another critical density associated with the mechanical elimination of abnormal cell clusters.

We examined the dynamics for different sets of mechanical parameters (Λ,Γ) and found that the phase diagrams for elimination success/failure were similar for the sets of mechanical parameters giving the same value of bulk modulus *K*(Λ,Γ) (Fig 2G). This indicates that tissue physical properties determine which density, i.e., ρ* or ρ**, is critical for the mechanical elimination of abnormal cell clusters. It should be noted that none of the simulation results changed qualitatively when a small amount of noise was added to the system (e.g., the one obeying the Ornstein–Uhlenbeck process [46] to the coefficient of line tension Λ).

### Mechanical homeostatic cell density

**Fig 3:**
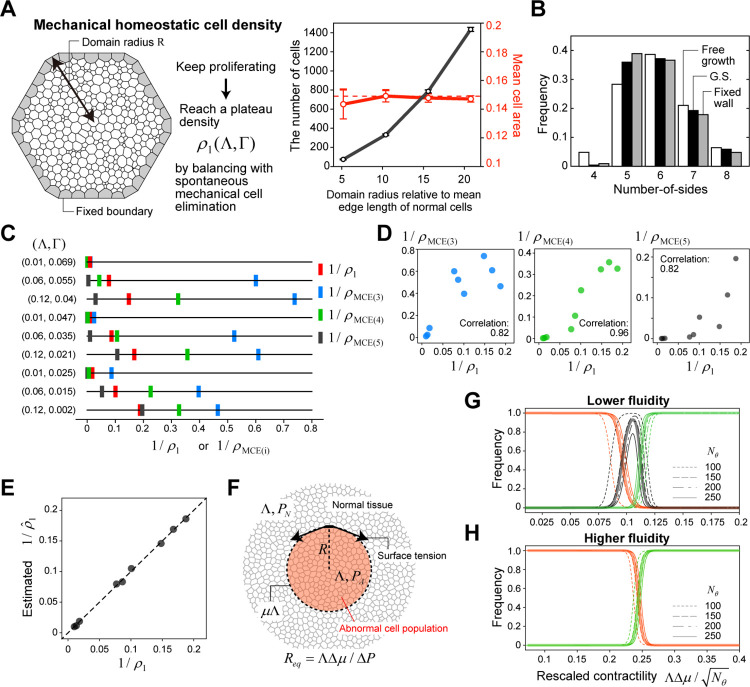
Mechanical homeostatic cell density and scaling of phase diagrams for elimination success/failure. (A) The simulation settings in which cells continue proliferating within a restricted space with a fixed boundary (gray cells; left). The dependency of the number of cells in the domain (black) and mean cell area (red) on the domain size R (right). Each open circle indicates the temporal average of a single simulation run. The error bars indicate the standard deviation over time. (B) The distribution of the number of cell sides within an abnormal cell cluster in the growth suspension phase (black) and in a growing tissue composed of a single cell type under free boundary (white) and fixed boundary (gray) conditions. These histograms were obtained by the temporal average over a single simulation run. (C) The reciprocal values of mechanical homeostatic cell density *ρ*_1_ (red) and the upper limit of surrounding cell density below which a regular polygon with *i* edges can exist (*ρ*_MCE(*i*)_, *i* = 3, blue; *i* = 4, green; *i* = 5, black) for different sets of mechanical parameters (Λ,Γ). (D) Pairwise plots of 1/*ρ*_1_ and 1/*ρ*_MCE(*i*)_ (*i* = 3, left; *i* = 4, middle; *i* = 5, right) showing their positive correlations. (E) Comparison of the values of *ρ*_1_ and the multiple linear regression results ${\hat{\rho }}_{1}$ for the nine different mechanical parameter sets (see also Eq (1)). The broken line represents ${\hat{\rho }}_{1}$ = *ρ*_1_. (F) A schematic diagram for the 2D Laplace’s law. At the interface, the energy is higher by ΛΔ*μ* = Λ(*μ*−1) per unit edge length. *P*_*N*_ and *P*_*A*_ are the pressure within normal and abnormal cell populations, respectively, the difference in which is denoted by Δ*P* = *P*_*A*_ − *P*_*N*_. *R*_*eq*_ is the radius of the abnormal cell cluster (denoted by *R*) at mechanical equilibrium. (G, H) The frequencies of elimination failure (red), growth suspension (black), and elimination success (green) against the rescaled contractility $\Lambda \Delta \mu /\sqrt{N_{\theta }}$ for the tissues with lower fluidity (G) or higher fluidity (H). The thin curves show the results (fitted by the Hill functions) for *N*_*θ*_ = 100, 150, 200, and 250, and the thick curves show the approximations using the Hill functions for all of the simulation data with four different values of *N*_*θ*_. In (A), (B), and (G), the parameter set (Λ,Γ = 0.12, 0.04) was used. In (C), (D), and (E), the nine parameter sets in Fig 1B were used. In (H), the parameter set (Λ,Γ = 0.01, 0.025) was used.

In the growth suspension phase for tissues with lower fluidity, a balance between the increase in the number of cells due to cell division and the decrease due to mechanical cell elimination was observed, while keeping a unique density ρ* for a given set of (Λ,Γ). This balance is considered to be equivalent to the concept of homeostatic density, defined as the cell density with a steady state between cell division and apoptosis, introduced earlier by Basan et al. [27]. To date, signaling pathways for density-dependent regulation of cell division or apoptosis have been reported in some experimental studies [15–17,19,20,47], and the threshold of this regulation is considered to determine the homeostatic density. Importantly, in our model, we did not explicitly introduce such a density threshold (note that the T2 threshold is much smaller than 1/ρ* or 1/ρ** and is not related to the focal density). In the growth suspension phase, as our previous study suggested, cell elimination is thought to occur spontaneously due to mechanical instability via saddle-node bifurcation when mechanical conditions allowing each cell to exist stably with a finite size are violated due to a density increase caused by cell division [31]. In fact, when cells continue to proliferate within a fixed wall (Fig 3A, left), they reach a plateau density specific to their mechanical parameters regardless of the size of the restricted space (Fig 3A, right). We call this mechanical homeostatic cell density *ρ*_1_(Λ,Γ), as a subtype of homeostatic density defined previously. We confirmed that the value of *ρ*_1_ was in close agreement with the density in the growth suspension phase ρ*, i.e., ρ* ≈ *ρ*_1_ (Fig 2D).

At present, it is difficult to determine the exact value of *ρ*_1_ from the physical properties, but it is possible to estimate it by statistical regression as follows. According to Lewis’s law [48], the average size of a polygonal cell is highly correlated with its number of sides. In a proliferating cell population in the growth suspension phase or within a fixed wall, an increase in cell number due to proliferation is balanced by mechanical cell elimination, and triangular and/or quadrilateral cells, which generally have smaller areas, are more likely to be eliminated compared with polygonal cells with a greater number of sides. For example, in the case of (Λ,Γ) = (0.12, 0.04), which corresponds to the physical properties of epithelial cells in the Drosophila wing disc [34], the distribution of the number of sides in simulations indicates that the frequency of quadrilaterals in the growth suspension phase (or growth within a fixed wall) is significantly smaller than that for a growing tissue composed of a single cell type under the free boundary condition (Fig 3B). Previously, we showed analytically that the conditions under which each cell can exist in a mechanically stable manner are determined by the number of sides of the focal cell and the density of surrounding cells [31]; the analytical solution of the upper limit of surrounding cell density below which a regular polygon with *i* edges can exist (denoted by *ρ*_MCE(*i*)_, where MCE stands for mechanical cell elimination) was obtained. The relationship *ρ*_MCE(*i*)_ < *ρ*_MCE(*j*)_ (*i* < *j*) holds, and the value of *ρ*_MCE(*i*)_ depends on the physical parameters (Λ,Γ) (Fig 3C). In the case of (Λ,Γ) = (0.12, 0.04), since the rate of elimination of quadrilaterals and the rate of proliferation of all abnormal cells are balanced under the growth suspension phase, *ρ*_1_ is expected to be bounded by the density at which pentagons, but not quadrilaterals, can exist stably, and the inequality *ρ*_MCE(4)_ < *ρ*_1_ < *ρ*_MCE(5)_ actually holds for *ρ*_1_ value obtained by the simulations (Fig 3C). For another parameter set, the elimination rate of triangles and the proliferation rate of all abnormal cells are balanced, and the inequality *ρ*_MCE(3)_ < *ρ*_1_ < *ρ*_MCE(4)_ holds true (e.g., (Λ,Γ) = (0.06, 0.055)) (Fig 3C). Interestingly, across different physical parameter sets, the values of *ρ*_1_ and *ρ*_MCE(*i*)_ (*i* = 3, 4, 5) are strongly correlated with each other (Fig 3D), and the following multiple linear regression gives a very good fit (Fig 3E):

$$\rho_{1}\approx \sum_{i=3}^{5}c_{i}\rho_{\mathrm{MCE}(i)},$$ (1)

where *c*_*i*_ are constants. Since *ρ*_MCE(*i*)_ can be derived analytically for a given set of (Λ,Γ), *ρ*_1_ can be predicted with high accuracy.

Regarding the critical density ρ** in tissues with higher fluidity, ρ** was unexpectedly different from *ρ*_1_ (i.e., ρ** ≠ *ρ*_1_) (Fig 2F), suggesting that there is another critical cell density other than mechanical homeostatic density associated with the mechanical elimination of abnormal cell clusters. In a later section, we will derive approximate analytical solutions for elimination conditions by mathematical analysis and will clarify the differences in the elimination mechanism depending on the physical properties of tissues and how the values of ρ*, ρ**, and corresponding critical contractility are estimated.

### Scaling of phase diagrams

Before addressing why the phase diagram differs depending on tissue physical properties, and how to determine the values of critical cell density and critical contractility necessary to achieve abnormal cell cluster elimination, we examined the dependence of phase diagram on the initial cluster size *N*_*θ*_. To achieve elimination success, the pressure difference between abnormal and normal tissues generated by interfacial tension derived from the relative contractility Δ*μ* (= *μ*−1) must overcome the expansion of abnormal cells via proliferation. According to Laplace’s law, in the case of the vertex dynamics model, this pressure difference between the two tissues (denoted by Δ*P*) is given by ΛΔ*μ*/*R*, where *R* is the radius of the abnormal cell population (Fig 3F). Assuming that the square root of the number of abnormal cells at the onset of the contraction force, *N*_*θ*_, corresponds to *R*, the phase diagram for elimination success/failure is expected to scale with $\Lambda \Delta \mu /\sqrt{N_{\theta }}$. Fig 3G and 3H show the superimposed diagrams obtained by simulations for different *N*_*θ*_ values after rescaling the horizontal axis, demonstrating that the scaling relationship holds well. This scaling relationship was confirmed for different physical parameter sets (Fig 3G for a solid tissue and Fig 3H for a fluid tissue).

### Mathematical analysis for the derivation of analytical solutions

**Fig 4:**
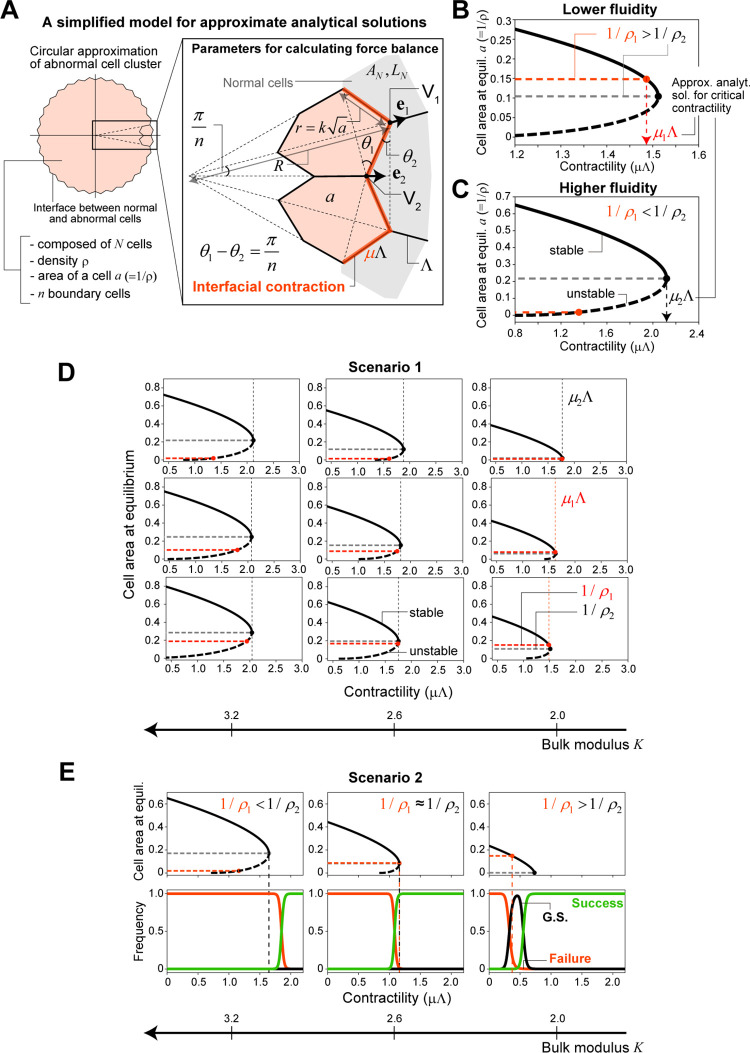
Mathematical analysis for deriving analytical solutions. (A) A simplified model for deriving approximate analytical solutions for elimination conditions (see main text for details). (B, C) Dependency of the cell area *a* (= 1/*ρ*) at equilibrium and its local stability (thick black curve) on interfacial contractility *μ*Λ obtained by our approximate analytical solutions for tissues with lower fluidity (B) or higher fluidity (C) under Scenario 1. There are two types of characteristic cell density that can be critical for the mechanical elimination of abnormal cell clusters; one is mechanical homeostatic density (*ρ*_1_, red horizontal broken line), and the other is related to mechanical stability as a population (*ρ*_2_, gray). For tissues with lower fluidity (B), the inequality *ρ*_2_ > *ρ*_1_ holds. Thus *ρ*_1_ is reached first when *μ* increases and functions as a critical density for mechanical elimination, where the corresponding contractility is denoted by *μ*_1_Λ. On the other hand, for tissues with higher fluidity (C), *ρ*_2_ < *ρ*_1_ holds, and *ρ*_2_ functions as the critical density, for which the corresponding contractility is *μ*_2_Λ. (D) Dependency of the cell area at equilibrium and its local stability on interfacial contractility for different sets of mechanical parameters under Scenario 1 (thick solid/dotted curves). The red and black vertical dotted lines represent *μ*_1_Λ and *μ*_2_Λ, respectively, and are the same as those in Fig 2G, showing that the derived analytical solution explains the simulation results well. In theory, when *ρ*_2_ > *ρ*_1_, a growth suspension phase is expected. (E) The results of a similar analysis under Scenario 2; (bottom) phase diagram from the simulations and (top) analytical solutions. When the tissue fluidity is lower, the differences between *ρ*_1_ and *ρ*_2_ and between *μ*_1_Λ and *μ*_2_Λ are more marked. In (B) and (C), the parameter sets (Λ,Γ) = (0.12, 0.04) and (Λ,Γ) = (0.01, 0.025) were used, respectively. In (D), the nine parameter sets shown in Fig 1B were used. In (E), the three parameter sets (Λ,Γ) = (0.01, 0.025), (0.06, 0.035), and (0.12, 0.04) were used. All results were calculated for *N*_*θ*_ = 100.

To better understand the observations from numerical simulations, we attempted to derive the approximate analytical solutions for elimination conditions. Let us consider the following simplified situations (Fig 4A):

(i) The abnormal cell cluster is composed of *N* cells and has a rotationally symmetric circular shape with density *ρ*. All abnormal cells have the same area *a* = 1/*ρ*, do not proliferate, and are in mechanical equilibrium under a given contractility *μ*Λ.
(ii) At the interface, *n* abnormal cells are in contact with the surrounding normal tissue, and they are congruent hexagons with a perimeter of $6k\sqrt{a}$, where *k* is a constant. Each edge length between abnormal and normal cells (*r*) is $k\sqrt{a}$. All normal cells are axial symmetric with respect to the radial axis through the center of the abnormal cell cluster, and they have the same area *A*_*N*_ and perimeter *L*_*N*_.
(iii) When *μ* changes, only *ρ* (or *a*) changes, and the values of the other quantities (*N*, *n*, *k*, *A*_*N*_, *L*_*N*_, *θ*_1_) do not change (see Fig 4A for the definition of angle *θ*_1_).
  Under these situations, we obtained the following force balance equation (see Methods for the derivation):
$${\sqrt{a}}^{3}-3q_{1}\sqrt{a}+2q_{2}=0,$$ (2A)
$$q_{1}=\frac{A_{N}}{3}-2\Gamma (1+\frac{\pi }{n}),$$ (2B)
$$q_{2}=\frac{\pi }{2nk}\mu \Lambda -\Gamma L_{N}(\frac{n-\pi }{2nk}).$$ (2C)
  Since Eq (2) is the cubic equation of $\sqrt{a}(=1/\sqrt{\rho })$, the area or density of abnormal cells can be solved analytically for a given contractility *μ*Λ. When Eq (2) has finite positive real solutions, the abnormal cell cluster can exist; otherwise, the cluster disappears.

To calculate the roots of this equation, let us make the following additional assumptions about the quantities *k*, *n*, *A*_*N*_, and *L*_*N*_:

(iv) The shape of each interfacial abnormal cell is close to a regular hexagon, $k\approx \sqrt{2\sqrt{3}}/3$.
(v) Regarding *n*, the relationship $n\approx \sqrt{2\sqrt{3}\pi N}$ holds under assumption (iv) and for large *N* (see Methods). Here, we adopted *N*_*θ*_ as the value of *N*.
(vi) The area and perimeter in the ground state in Eq (6) (see Methods) are good candidates for those of surrounding normal tissues, *A*_*N*_ and *L*_*N*_. Note that we focused only on the parameter region in which the ground state is given as the regular hexagonal packing [34,49]. Then, the relationship $A_{N}=(\sqrt{3}/24)L_{N}^{2}$ holds, where *L*_*N*_ is the root of $L_{N}^{3}+8(12\Gamma -\sqrt{3})L_{N}+48\Lambda =0$ [34,49].

Fig 4B and 4C show the dependency of the cell area *a* (= 1/*ρ*) at the equilibrium of Eq (2) and its local stability on *μ*Λ for a solid tissue with (Λ,Γ) = (0.12, 0.04) and a fluid tissue with (Λ,Γ) = (0.01, 0.025), respectively (see also Methods). With the change in *μ*, the number of equilibria varies. For a wide range of mechanical parameters, there is a bifurcation point at which positive real roots disappear (the black closed circle in Fig 4B); more rigorously, the bifurcation point appears when the tissue has a physical property for which *q*_1_ > 0 is satisfied in the cubic equation, Eq (2). We denote the area/density and relative contractility at this bifurcation point as *a*_2_ (or *ρ*_2_) and *μ*_2_Λ, respectively, and those values can be given analytically as follows:

$$\rho_{2}=\frac{1}{q_{1}}=\frac{1}{A_{N}/3-2\Gamma (1+\pi /n)},$$ (3A)

$$\mu_{2}\Lambda =k\frac{2n}{\pi }{\left(\frac{A_{N}}{3}-2\Gamma (1+\frac{\pi }{n})\right)}^{3/2}+\Gamma L_{N}(\frac{n}{\pi }-1).$$ (3B)

Unlike mechanical homeostatic density (*ρ*_1_), which is determined only by the cell mechanical parameters (Λ,Γ), *ρ*_2_ depends on *n* ($\propto \sqrt{N}$); note that *A*_*N*_ can be approximated as a function of (Λ,Γ) according to the above condition (vi). However, when the cluster size of abnormal cells is large enough, the effect of the term that includes *n* can be neglected, and *ρ*_2_ is also determined solely by the cell mechanical parameters. Importantly, for tissues with higher fluidity (or larger bulk modulus *K*), this density with respect to mechanical stability as the cell population *ρ*_2_ is in close agreement with ρ** observed in the simulations under Scenario 1 and is lower than the mechanical homeostatic cell density *ρ*_1_; i.e., *ρ*_2_ ≈ ρ** < *ρ*_1_ (Figs 2F and 4C). In contrast, for the tissues with lower fluidity, *ρ*_2_ was larger than, and not consistent with, ρ* (≈ *ρ*_1_) (Figs 2D and 4B).

These results can explain the qualitative difference in the phase diagram depending on the tissue physical properties observed in the simulations (Figs 2G and 4D). For a given physical property, *ρ*_1_(Λ,Γ) and *ρ*_2_(Λ,Γ) are determined. Since the physical property determines which of these is smaller, the one that is reached first differs when *μ* increases; when *ρ*_2_ < *ρ*_1_, *ρ*_2_ is the critical density for the elimination of abnormal cell clusters, and when *ρ*_2_ > *ρ*_1_, mechanical homeostatic cell density *ρ*_1_ becomes the critical density. Intuitively, when tissue fluidity is higher, the bulk modulus is larger and the shear modulus smaller, rendering it easier for individual cells to deform but more difficult for them to alter their volume. Therefore, when the abnormal tissue is compressed, the mechanical stability as a cell population becomes the criterion for elimination. For this reason, *ρ*_2_ can be interpreted as the critical density. On the other hand, when tissue fluidity is lower, it is easier for individual cells to change their volume. Thus, when the interfacial contractility increases, the mechanical stability of individual cells becomes critical for elimination. For this reason, *ρ*_1_, which is strongly correlated with *ρ*_MCE(*i*)_ (*i* = 3, 4, 5), as shown in Fig 3D, is the critical density.

The values of critical density and contractility necessary to achieve mechanical elimination of abnormal cell clusters differ depending on the scenario. Fig 4E shows the phase diagram obtained from simulations under Scenario 2 and the analytical solutions; it should be noted that under Scenario 2, condition (vi) was somewhat of an overestimation for the values of *A*_*N*_ and *L*_*N*_ probably because the abnormal cell density at the onset of the interfacial contraction was higher than that under Scenario 1. Thus we adopted the following modified condition (vi’) for calculation of *ρ*_2_ and *μ*_2_Λ:

(vi’) The values of the area and perimeter of the interfacial normal cells (*A*_*N*_ and *L*_*N*_, respectively) are estimated by solving the force balance equation Eq (2) when *μ* = 1; i.e.,

$$A_{N}+\frac{\Gamma (n-\pi )}{nk\sqrt{a_{0}}}L_{N}-a_{0}-\frac{6\Gamma (n+\pi )}{n}-\frac{\pi \Lambda }{nk\sqrt{a_{0}}}=0,$$ (4)

where *a*_0_ is the mean area of abnormal cells just before the onset of interfacial contraction, and its value from the simulations was used. In addition, $A_{N}=(\sqrt{3}/24)L_{N}^{2}$ was also assumed as in condition (vi).

As shown in Fig 4E, the critical density/contractility predicted by the analytical solution was in good agreement with the simulation. Expectedly, the contraction force required for elimination was smaller under Scenario 2 than under Scenario 1 for a given set of (Λ,Γ). One of the major differences in the results under Scenario 1 versus Scenario 2 is the gap between *ρ*_1_ and *ρ*_2_ and the disappearance of the bifurcation point in Scenario 2 for solid tissues (Fig 4E, right). This can be understood by Eqs (2B) and (3A); under Scenario 2, *A*_*N*_ is smaller and *ρ*_2_ larger; especially, when *A*_*N*_ is less than 6Γ(1+*π*/*n*), *q*_1_ becomes negative, and the bifurcation point disappears.

On the other hand, the force balance equation can also explain the scaling of the phase diagram observed in the simulations. Assuming that *n* ($\propto \sqrt{N}$) is sufficiently large (specifically, *n*≫*π*), for simplicity, Eq (2) can be rewritten as follows:

$$\frac{(\Delta \mu )\Lambda }{n}=\frac{(\mu -1)\Lambda }{n}\approx \frac{\mu \Lambda }{n}\propto \frac{\mu \Lambda }{\sqrt{N}}\propto -{\sqrt{a}}^{3}+(A_{N}-6\Gamma )\sqrt{a}+\frac{\Gamma L_{N}}{k}.$$ (5)

Thus, the value of $\Lambda \Delta \mu /\sqrt{N_{\theta }}$ determines the success or failure of mechanical elimination of abnormal cells.

### Effects of the relative proliferation rate and the difference in physical properties between normal and abnormal cells

Here, we show some other findings obtained from the simulations, in which Scenario 2 was used because it reflects more realistic situations in a biological sense.

#### (i) Abnormal cells are eliminated more easily when their relative proliferation rate is higher

**Fig 5:**
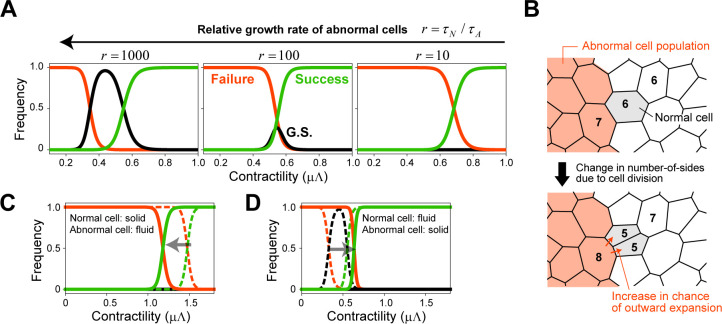
Effects of the relative proliferation rate and the difference in physical properties between normal and abnormal cells. (A) The dependence of the relative growth rate of abnormal cells (*r*) on the phase diagram for elimination success. Simulations were performed under Scenario 2 and *N*_*θ*_ = 100. (B) When a cell divides, its daughter cells have fewer sides on average compared with the mother cell, while the number of sides of the two cells adjacent to the divided cell increases. As there is a positive correlation between the number of cell sides and the average cell size (Lewis’s law), the areas of the adjacent cells increase. Thus, division of normal cells at the interface could be a mechanism for increasing the likelihood of outward expansion of abnormal cell populations. (C, D) The effects of different physical properties between normal and abnormal cells on the phase diagram. When the abnormal cells have a fluidity higher than that of the surrounding normal cells, the critical contractility required for elimination of an abnormal cell cluster becomes smaller than that when normal cells have the same physical property as abnormal cells, and vice versa. The broken lines show the case in which both normal and abnormal cells are fluid (C) or solid (D).

In the above simulations, it was assumed that the normal cell cycle time was sufficiently long and negligible compared with that of the abnormal cell cycle time. Here, we explicitly considered the proliferation of normal cells and examined how the elimination success rate depends on the relative growth rate of abnormal cells to normal cells, *r* = (1/*τ*_A_)/(1/*τ*_N_) = *τ*_N_/*τ*_A_. Note that we modulated the relative proliferation rate by changing the cell cycle duration of normal cells while leaving that of abnormal cells unchanged. Fig 5A shows the phase diagram for *r* = 1000, 100, and 10 for (Λ,Γ) = (0.12, 0.04). Interestingly, when surrounding normal cells proliferate, and the relative proliferation rate of abnormal cells decreases, the abnormal cells become difficult to eliminate. That is, slower proliferation of abnormal cells promotes their growth as a population. This might be counterintuitive but can be explained as follows. When a cell divides, its daughter cells have on average fewer sides compared with the mother cell [50,51]. For example, a hexagonal cell divides into two pentagonal daughter cells in general (Fig 5B). Simultaneously, the two cells adjacent to the divided cell exhibit an increase in their number of edges (Fig 5B). As stated, the number of cell edges and the average cell size are positively correlated, referred to as Lewis’s law [48]. Therefore, the size of the adjacent cells that obtained new edges tends to increase. When normal cells proliferate at a high frequency (i.e., when the relative growth rate of abnormal cells is low), division of normal cells at the interface increases the number of sides and the size of the adjacent abnormal cells; this leads to an increased likelihood of outward expansion of the abnormal cell population and also acts as a perturbation at the interface, rendering it difficult to maintain the growth suspension phase (Fig 5B). Conversely, when normal cells do not proliferate, or their proliferation is sufficiently slow, abnormal cells lose their edges and areas at interfaces via cell division and are pushed inward by normal cells, resulting in higher density. As mentioned earlier, the condition for stable existence of each polygonal cell depends on the density or the average size of its surrounding cells. In high-density cell populations, individual cells are sensitive to fluctuations in size due to the T1 process and/or division of surrounding cells, and the probability of them losing their stable equilibrium and disappearing via saddle-node bifurcation increases [31]. In a related study of the number of cell sides, Tsuboi et al. addressed the issue of which cell population fills the gap when cell death occurs at the interface between two proliferating cell populations [52]. Thus, for abnormal cells, rapid growth is not always an advantage.

#### (ii) Effects of differences in physical properties between normal and abnormal cells

Above, we analyzed cases in which abnormal and normal cells have the same physical properties. However, there are known cases in which the physical properties differ between mutant and normal cells [26,53,54]. Thus, we investigated the effects of different physical properties between normal and abnormal cells on the elimination of abnormal cell clusters. Fig 5C shows a phase diagram for the case when abnormal cells are more fluid (Λ_A_,Γ_A_ = 0.12, 0.01) and the surrounding normal cells more solid (Λ_N_,Γ_N_ = 0.12, 0.04). The critical contractility *μ*Λ required for the elimination of abnormal cell clusters was somewhat smaller than (but not much different from) that in the case in which normal cells have the same physical properties as abnormal cells. This difference can be explained as follows: when normal cells are solid, the large shear modulus renders the T1 process less likely to occur at the interface, and the abnormal cells are trapped within the normal cells, increasing their density and facilitating their elimination. On the other hand, Fig 5D shows the phase diagram when abnormal cells are solid (Λ_A_,Γ_A_ = 0.12, 0.04) and normal cells are more fluid (Λ_N_,Γ_N_ = 0.12, 0.01). The major difference from the case in which both cell types are solid is that the growth suspension phase was not observed. This is because the T1 process occurs more readily when the surrounding normal cells are fluid due to a smaller shear modulus, thus acting as a perturbation to the topology of interfacial abnormal cells and rendering it difficult to maintain the size of an abnormal cell population constant for longer. This also makes it more difficult to confine abnormal cells, leading to lower cell densities and an increase in the critical contractility *μ*Λ required for elimination success, compared with the case in which normal cells have the same physical properties as normal cells (i.e., solid).

## Discussion

In this study, motivated by experimental observations, we theoretically evaluated the conditions necessary for the elimination of a growing abnormal cell population by mechanical contraction at the interface between normal and abnormal cell populations. The main result of this study is the numerical and analytical demonstration that there are two types of critical densities for mechanical elimination of abnormal cell populations, and both depend on tissue physical properties. One of these critical cell densities, *ρ*_1_, is related to the homeostatic density previously introduced by Basan et al. [27]. However, we did not explicitly assume dependence of the abnormal cell proliferation rate on density, and the ability of the abnormal cell population to maintain a constant density *ρ*_1_ even as it continues to proliferate is attributed to the spontaneous mechanical elimination of each cell. In this sense, *ρ*_1_ is different from the original definition of homeostatic density, which explicitly assumes the dependence (i.e., threshold) of cell proliferation or death on density, and here we refer to *ρ*_1_ as mechanical homeostatic cell density, the value of which is determined by cell mechanical parameters only. The other critical cell density, *ρ*_2_, is a quantity related to the mechanical stability as a population, and it satisfies the cubic equation as shown in the force balance equation, Eq (2). Note that Eq (2) has the same form as that of the force balance equation for a single cell with interfacial contractility between adjacent cells, which was analyzed in our previous study [31]. For a given physical property of cells, when *ρ*_2_ > *ρ*_1_, *ρ*_1_ is first reached as *μ* increases, providing the critical cell density required for mechanical elimination of the abnormal cell population, and vice versa. From the theoretical side, how the concepts of critical density developed here can be meaningfully generalized to a tensorial quantity (such as homeostatic stress tensor [55]) remains an important issue.

We also found counterintuitive properties, such as a negative correlation between the proliferation rate of abnormal cells and the likelihood of their expansion by escaping elimination. As might be obvious, in some cases of cell competition, the fast-growing cell populations win [21]. However, our results indicate that when contractile forces act on the interface between two cell populations (i.e., *μ* > 1), rapid growth is not always an advantage. This would be an interesting point that can be verified experimentally. For example, in some experimental systems used to evaluate cell competition, it would be feasible to examine the effect on the elimination efficiency of abnormal cells when their relative growth rate is decreased by increasing the growth rate of normal cells, as we did in our simulation. In addition, it may be possible to demonstrate experimentally that the phase diagram for elimination success/failure can vary depending on the tissue physical properties, as shown by our simulations (Fig 2B and 2E). For example, the stiffness of human mesenchymal stem cells (hMSCs) depends on the substrate rigidity [56], and the stiffness of MDCK cells in the confluent state depends on the number of cells seeded [57]. Furthermore, it is also possible to induce local actomyosin activity and to control its intensity, in principle, using recent optogenetic techniques, which enable drawing a phase diagram by changing the contractility at the interface between normal and abnormal tissues.

The inability to describe the 3D effects of the cell elimination process is a limitation of our model. In our 2D model, the elimination of abnormal cells is indicated by their disappearance from the apical plane. However, as Bielmeier et al. reported experimentally, when the size of an abnormal cell population increases, it does not lose its apical surface but instead invaginates along the apico-basal plane and is eliminated after forming a cyst [14]. Extending our model to a 3D version might reveal conditions for mechanical cell elimination via apical exclusion and invagination followed by cyst formation, respectively.

In the current study, we assumed that the surrounding normal cells recognize the emergence of abnormal cells and that the contractile force at the interface between normal and abnormal cells increases once the abnormal cells reach a certain population size (in the case of Scenario 2). In real biological situations, some aspects of the signal transduction system involved in cell competition are known, although the recognition mechanisms are not wholly understood. Changes in the mechanical as well as chemical environment caused by the emergence of abnormal cells may provide a clue to their recognition. The possibility of mechanical recognition of abnormal cells would be an interesting issue both theoretically and experimentally to explore in the future.

## Methods

### A vertex dynamics model

In brief, in vertex dynamics models, each cell is represented as a polygon formed by linking several vertices, and the motion of each vertex obeys the following equations (non-dimensionalized version) [37]:

$$\frac{dr_{i}}{dt}=-\frac{\partial U}{\partial r_{i}},$$ (6A)

$$U=\sum_{\alpha }\frac{1}{2}(A_{\alpha }-1{)}^{2}+\sum_{<\alpha ,\beta >}\Lambda_{\alpha \beta }l_{\alpha \beta }+\sum_{\alpha }\frac{\Gamma_{\alpha }}{2}{L_{\alpha }}^{2},$$ (6B)

where **r**_*i*_ is the positional vector of the *i*-th vertex. The first term of the energy function *U* indicates an area constraint, where *A*_*α*_ is the area of cell *α*. The second term represents the line tension and cell–cell adhesion at the edges between cell *α* and cell *β*, where *l*_*αβ*_ is the edge length, and Λ_*αβ*_ is the coefficient of line tension. The third term represents the perimeter elasticity, where *L*_*α*_ is the perimeter of cell *α*, and Γ_*α*_ is the coefficient of perimeter elasticity. Λ and Γ determine the physical properties of a cell population or tissue. Quantitatively, the bulk or shear modulus (denoted by *K* or *G*, respectively) is an index characterizing the physical properties of the tissue and, in the case of the above vertex dynamics model, is given as follows [34,49]:

$$K=9\sqrt{3}{\left(l_{g}(\Lambda ,\Gamma )\right)}^{2}+8\sqrt{3}\Gamma -2,$$ (7A)

$$G=12\sqrt{3}\Gamma +\sqrt{3}\Lambda /l_{g}(\Lambda ,\Gamma )=3(1-A_{g}(\Lambda ,\Gamma )),$$ (7B)

where *l*_*g*_(Λ,Γ) and *A*_*g*_(Λ,Γ) are the cell edge length and cell area at the ground state, respectively, with ground-state networks defined as the polygon configurations minimizing the energy function *U* for given physical parameters (Λ,Γ) within an infinite plane [34,49] (see Table 1 for the parameter values used in this study).

**Table 1:** Descriptions of the parameter values used in our simulations.

Parameter	Description	Value
(Λ, Γ)	Coefficient of line tension (Λ) and perimeter elasticity (Γ)	(0.12,0.04), (0.01,0.025), (0.01,0.047), (0.01,0.069), (0.06,0.015), (0.06,0.035), (0.06,0.055), (0.12, 0.015), (0.12,0.021)
$\theta_{T1}/\bar{l}$	T1 transition length threshold	0.01–0.05
$\theta_{T2}/\bar{A}$	T2 transition area threshold	0.01–0.05
*Δt*	Time step	0.0001
*τ*_A_	Cell cycle duration of abnormal cells	5, 25, 50, 100
*τ*_N_	Cell cycle duration of normal cells	inf., 50, 500, 5000
*b*	Growth rate of the target cell area during the mitotic phase	10
*δA*	Constant increment in target cell area for the adder division rule	0.15 or 0.2

As a consequence of push–pull dynamics among cells in growing tissues, cellular rearrangement occurs. This rearrangement is driven by edge reconnections (T1 process) and occurs when the edge length is less than the T1 threshold *θ*_T1_ [34,37]. Cell elimination occurs simply by removing a cell with an area less than the T2 threshold *θ*_T2_ [31]. We mainly used $\theta_{T1}/\bar{l}$ = $\theta_{T2}/\bar{A}$ = 0.01, where $\bar{l}$ is the average length of normal cells and $\bar{A}$ is the average cell area of normal cells. We confirmed that the changes in the values of *θ*_T1_ and *θ*_T2_ had minor effects on our results; specifically, the relationships ρ* ≈ *ρ*_1_ and ρ** ≈ *ρ*_2_ still hold, although the mechanical homeostatic cell density (*ρ*_1_) changed slightly depending on *θ*_T1_ and *θ*_T2_ (please see below or the text for the definitions of *ρ*_1_, *ρ*_2_, ρ*, and ρ**) (S2 and S3 Figs).

In the simulations, the Euler method was used with the time step *Δt* = 0.0001, corresponding to 1/50000 of the cell cycle time, which is sufficiently small based on a previous study by Kursawe et al. [58]. As for boundary conditions, we tested both free and fixed boundary conditions and confirmed that the simulation results are qualitatively the same under both conditions; in this study, we show the simulation results under the free boundary condition.

### Model for the cell division

We introduced a clock representing the cycle for each cell. The clock progresses linearly with time. When the timer reaches a threshold (*τ*_A_ for abnormal cells and *τ*_N_ for normal cells), the cell enters the mitotic phase of division. To prevent the synchronization of cell division, we included ± 20% randomness (uniformly distributed) into the cell cycle time. In most simulations, we used *τ*_A_ = 5 and *τ*_N_ = infinity. As shown in S4 Fig, the intensity of the interfacial contractility necessary for eliminating abnormal cell clusters depends somewhat on the cell cycle time, but the phase diagram for elimination success/failure converges to a certain profile with increasing cell cycle time. For tissues with lower fluidity, the relationship ρ* ≈ *ρ*_1_ holds well. For tissues with higher fluidity, the difference between ρ** and *ρ*_2_ becomes larger for larger *τ*_A_, but both ρ** and *ρ*_2_ still show close values. During the mitotic phase, the target cell area increases linearly with time and thus the first term of Eq (6B) is replaced by (*A*_*α*_ − (1 + *bt*))^2^ / 2, where *b* is the growth rate of the target area, and we used *b* = 10. When the actual area of the focal cell (*A*_*α*_) doubles, the cell divides with an axis through its center, and the cell cycle clock is reset to zero. A recent study of the cell cycle suggested that some epithelial cells divide by adding a constant volume between consecutive division events [59]. Thus, we also tested this model, called the adder model; specifically, a cell divides when the actual area of the cell (*A*_*α*_) becomes *A*_*α*_ + *δA*, where *δA* is a fixed increment, rather than doubling (i.e., 2 *A*_*α*_). We confirmed that this change in the rule of cell division has a very minor impact quantitatively on our results (S5 Fig).

Regarding cell division orientation, we performed simulations under the following two cases: (i) the orientation is determined randomly from the uniform distribution, and (ii) the division plane is perpendicular to the cell longitudinal axis (i.e., Hertwig’s law [60]). Since there was no qualitative difference in the results between these cases, we present simulation results for case (i).

### Definitions of characteristic densities

Since multiple characteristic densities appear in this paper, we summarize them here. For each simulation run, the density of an abnormal cell population at time *t*, *ρ*(*t*), was defined as $\rho (t)\equiv N(t)/\sum_{i}A_{i}(t)$, where *A*_*i*_(*t*) and *N*(*t*) are the area of the *i*-th abnormal cell and the total number of abnormal cells, respectively. For each of these time profiles of density (S1B Fig), $\bar{\rho }$, defined by $\bar{\rho }\equiv \underset{t}{min}E_{\tau_{A}}[\rho (t)]$, was used as a quantity representing the time average of the density after the initial response to the interfacial contractile force, where *E*_*τ*A_[] represents a moving average with a *τ*_A_ time window. In the case of elimination success, $1/\bar{\rho }$ was defined as zero.

In the simulations for tissues with lower fluidity, a growth suspension phase was observed when the contractile force at the interface between abnormal and normal cells was moderate. In the growth suspension phase, the density was constant and depends on the cell mechanical parameters. This density observed in the simulation is called ρ*; mathematically, 1/ρ* is defined as the population average of $1/\bar{\rho }$ for each sample path over all simulation runs showing the growth suspension phase, i.e., 1/ρ* ≡ <$1/\bar{\rho }$>_G.S._ We showed that ρ* was in close agreement with mechanical homeostatic density (denoted as *ρ*_1_), which is defined as the plateau density when cells continue to proliferate within a fixed wall. The value of *ρ*_1_ depends on cell mechanical parameters but is independent of the size of the restricted space.

In the simulations for tissues with higher fluidity, another characteristic density related to mechanical elimination of abnormal cell clusters was observed, which we called ρ**. ρ** was defined as the density under the critical contractile force (i.e., at the jump point). We found this density to be in good agreement with the density *ρ*_2_, which is responsible for the mechanical stability of the abnormal cells as a population, where the value of *ρ*_2_ was derived analytically.

### Derivation of force balance equation Eq (2) and stability of equilibria

We considered a simplified model for deriving the approximate analytical solutions for elimination conditions (see the main text for the detailed settings and Fig 4A). The radial forces applied to the two interfacial vertices V_1_ and V_2_ (denoted by **F**_1_ and **F**_2_, respectively) of a boundary abnormal cell are given as follows:

$$\begin{array}{l} \;F_{1}=F_{1}e_{1}=[(1-a)r\;\cos\;\theta_{1}-2\mu \Lambda \;\sin\;\theta_{1}-12\Gamma r\;\sin\;\theta_{1}\\ -(1-A_{N})r\;\cos\theta_{1}+\Lambda +2\Gamma L_{N}-2\Gamma L_{N}\;\sin\theta_{1}]e_{1}, \end{array}$$ (8A)

$$\begin{array}{l} \;F_{2}=F_{2}e_{2}=[(1-a)r\;\cos\;\theta_{2}-\Lambda +12\Gamma r\;\sin\;\theta_{2}-12\Gamma r\\ -(1-A_{N})r\;\cos\;\theta_{2}+2\mu \Lambda \;\sin\;\theta_{2}+2\Gamma L_{N}\;\sin\;\theta_{2}]e_{2}, \end{array}$$ (8B)

where **e**_1_ and **e**_2_ are the unit vectors in the radial directions at V_1_ and V_2_, respectively (see Fig 4A and the main text for the definitions of *a*, *r*, *R*, Λ, Γ, *μ*, *A*_*N*_, *L*_*N*_, *θ*_1_, and *θ*_2_). When the force is balanced in each vertex within the abnormal cell population, the radial component (along the direction of **e**_1_) of the net force (denoted by *F*) applied to the focal abnormal cell on the interfacial side with normal cells is calculated as

$$F=F_{1}+F_{2}\;\cos\frac{\pi }{n}=F_{1}+F_{2}(1-\sin^{2}\frac{\pi }{2n}).$$ (9)

Using the sum-to-product formulas, the following relationships hold:

$$\cos\;\theta_{1}+\cos\;\theta_{2}=2B\;\cos\frac{\theta_{1}-\theta_{2}}{2}=2B\;\cos\frac{\pi }{2n},$$ (10A)

$$\sin\;\theta_{1}-\sin\;\theta_{2}=2B\;\sin\frac{\theta_{1}-\theta_{2}}{2}=2B\;\sin\frac{\pi }{2n},$$ (10B)

where *B* = cos((*θ*_1_+*θ*_2_)/2). In addition, for sufficiently large *n* and small *θ*_1_+*θ*_2_, the approximations cos(*π*/(2*n*)) ≈ 1, sin(*π*/(2*n*)) ≈ *π*/(2*n*), sin^2^(*π*/(2*n*)) ≈ 0, and *B* ≈ 1 hold. Then, the right-hand side of Eq (9) becomes

$$\begin{array}{l} F_{1}+F_{2}(1-\sin^{2}\frac{\pi }{2n})\\ =2B(A_{N}-a)r\;\cos\frac{\pi }{2n}-4B\mu \Lambda \;\sin\frac{\pi }{2n}-4B\Gamma (L_{N}+6r)\sin\frac{\pi }{2n}\\ +2\Gamma (L_{N}-6r)-F_{2}\;\sin^{2}\frac{\pi }{2n}\\ \approx 2\{(A_{N}-a)r-\frac{\pi \mu \Lambda }{n}-\frac{\pi \Gamma (L_{N}+6r)}{n}+\Gamma (L_{N}-6r)\}. \end{array}$$ (11)

Under the assumptions that the shape of the abnormal cell cluster is rotationally symmetric, and that interfacial abnormal cells become similarly smaller with the increase in *μ*, the radial coordinate of the vertex V_1_, *x* (= *R*), satisfies the following dynamics:

$$\begin{array}{l} \frac{dx}{dt}=\frac{dR}{dt}\propto \frac{d\sqrt{a}}{dt}\propto -F\\ \Leftrightarrow \frac{d\sqrt{a}}{dt}=-q_{0}\left\{{\sqrt{a}}^{3}-3(\frac{A_{N}}{3}-2\Gamma -2\Gamma \frac{\pi }{n})\sqrt{a}+\frac{\pi }{nk}\mu \Lambda -\Gamma L_{N}(\frac{n-\pi }{nk})\right\}\\ \Leftrightarrow \frac{d\sqrt{a}}{dt}=-q_{0}({\sqrt{a}}^{3}-3q_{1}\sqrt{a}+2q_{2}) \end{array}$$ (12)

where *πR*^2^ = *Na* was used in the first line; note that the number of abnormal cells in the cluster *N* is assumed to be constant in our model. *q*_0_ is a positive constant, and $r=k\sqrt{a}$ was used to obtain the equation in the second line. *q*_1_ and *q*_2_ are defined by Eqs (2B) and (2C), respectively. At equilibrium, Eq (2A) is obtained.

The number of equilibrium points in Eq (12) depends on the values of *q*_1_ and *q*_2_. Setting *f*(*x*) = − (*x*^3^ − 3*q*_1_*x* + 2*q*_2_), the stability of each equilibrium point $\sqrt{a^{*}}$ (= x*) is determined by the sign of the first derivative at the equilibrium point, i.e., *f’*(x*) = 3(*q*_1_ –(x*)^2^). When *q*_1_ > 0, the cubic equation *f*(*x*) has local minima/maxima, and the dynamics can have one or two non-negative equilibria depending on the value of *q*_2_. For *q*_2_ < 0, there is only one non-negative equilibrium x* >$\sqrt{q_{1}}$, and it is stable because *f’*(x*) < 0. For 0 < *q*_2_ <$q_{1}\sqrt{q_{1}}$, there are two equilibria. Denoting them as *x*_1_* and *x*_2_* (*x*_1_* > *x*_2_*), the larger one is stable and the smaller one unstable because *x*_1_* >$\sqrt{q_{1}}$ and *x*_2_* <$\sqrt{q_{1}}$. On the other hand, when *q*_1_ < 0, the cubic equation *f*(*x*) has no local minima/maxima, and when *q*_2_ < 0, the system has one equilibrium, which is stable because *f’*(x*) < 0.

### Approximating the relationship between *n* and *N*

Regarding the abnormal cell cluster as a circle of radius *R*, its perimeter is given by $2\sqrt{\pi Na}$, where *πR*^2^ = *Na* was used. In addition, when each abnormal cell at the interface takes a shape close to a regular hexagon, $a\approx 3\sqrt{3}r^{2}/2$ holds, and the perimeter is approximated as $\sqrt{6\sqrt{3}\pi N}r$. The perimeter length is also calculated by connecting the convex vertices (such as V_1_ in Fig 4A) of the interfacial abnormal cells, i.e., 2*nr*cos*θ*_2_. From the equality of both, the relationship $n=\sqrt{2\sqrt{3}\pi N}$ holds between *n* and *N*, where we assumed that interfacial abnormal cells are regular hexagons (i.e., *θ*_2_ = *π*/6).

## Supporting information

S1 Fig Dependence of 1/ρ* on the simulation time and the time courses of *ρ*(*t*). (A) The value of 1/ρ* was independent of the simulation time. (B) The time courses of cell density *ρ*(*t*) for different values of relative contractility *μ*. The cases of elimination failure (red), growth suspension phase (black), and elimination success (green) are shown. In (A) and (B), the parameter set (Λ,Γ = 0.12, 0.04) was used.
(EPS)

S2 FigDependence of the simulation results on *θ*_T1_.(A, D) Comparison of phase diagrams for elimination success/failure between the cases with $\theta_{T1}/\bar{l}$ = 0.01 and $\theta_{T1}/\bar{l}$ = 0.05 for tissues with lower fluidity (A) and higher fluidity (D). (B, E) The dependence of the average cell area ($1/\bar{\rho }$) on the interfacial contractility (*μ*Λ) for three values of $\theta_{T1}/\bar{l}$ for tissues with lower fluidity (B) and higher fluidity (E); left: 0.01; middle: 0.03; right: 0.05. Note that the mechanical homeostatic cell density (*ρ*_1_) depends slightly on the value of $\theta_{T1}/\bar{l}$ (see the text and Fig 3A for the definition of mechanical homeostatic density). (C) The difference between 1/ρ* and 1/*ρ*_1_ for different values of $\theta_{T1}/\bar{l}$ for tissues with lower fluidity. *ρ*_1_ was very close to ρ* for different values of $\theta_{T1}/\bar{l}$. (F) The difference between 1/ρ** and 1/*ρ*_2_ for different values of $\theta_{T1}/\bar{l}$ for tissues with higher fluidity. *ρ*_2_ was very close to ρ** for different values of $\theta_{T1}/\bar{l}$. In (A–C), (Λ,Γ = 0.12, 0.04) was used, and in (D–F), (Λ,Γ = 0.01, 0.025) was used.(EPS)

S3 FigDependence of the simulation results on *θ*_T2_.(A, D) Comparison of phase diagrams for elimination success/failure between the case with $\theta_{T2}/\bar{A}$ = 0.01 and that with $\theta_{T2}/\bar{A}$ = 0.05 for tissues with lower fluidity (A) and higher fluidity (D). (B, E) The dependence of the average cell area ($1/\bar{\rho }$) on the interfacial contractility (*μ*Λ) for three values of $\theta_{T2}/\bar{A}$ for tissues with lower fluidity (B) and higher fluidity (E); left: 0.01; middle: 0.03; right: 0.05. Note that the mechanical homeostatic cell density (*ρ*_1_) depends slightly on the value of $\theta_{T2}/\bar{A}$. (C) The difference between 1/ρ* and 1/*ρ*_1_ for different values of $\theta_{T2}/\bar{A}$ for tissues with lower fluidity. The difference converges to zero with the decrease in $\theta_{T2}/\bar{A}$. The reason why the difference becomes non-negligible as $\theta_{T2}/\bar{A}$ increases is that the density in the growth suspension phase decreases due to the death of larger cells. (F) The difference between 1/ρ** and 1/*ρ*_2_ for different values of $\theta_{T2}/\bar{A}$ for tissues with higher fluidity. *ρ*_2_ was very close to ρ** for different values of $\theta_{T2}/\bar{A}$. In (A–C), (Λ,Γ = 0.12, 0.04) was used, and in (D–F), (Λ,Γ = 0.01, 0.025) was used.(EPS)

S4 FigDependence of the simulation results on the cell cycle time *τ*_A_.(A, D) Comparison of phase diagrams for elimination success/failure among the cases with different cell cycle durations of abnormal cells for tissues with lower fluidity (A) and higher fluidity (D). The profile for elimination success/failure shifts depending on the cell cycle duration, but the phase diagram converges to a certain profile as the cell cycle duration increases. (B, E) The dependence of the average cell area ($1/\bar{\rho }$) on the interfacial contractility (*μ*Λ) for different cell cycle durations of abnormal cells for tissues with lower fluidity (B) and higher fluidity (E). (C) The difference between 1/ρ* and 1/*ρ*_1_ for different cell cycle durations for tissues with lower fluidity. *ρ*_1_ was very close to ρ* independently of cell cycle durations. (F) The difference between 1/ρ** and 1/*ρ*_2_ for different cell cycle durations for tissues with higher fluidity. For larger *τ*_A_, the difference was larger (but not significant). This is thought to be because the density of abnormal cells becomes slightly smaller when there is sufficient time for area relaxation after division. In (A–C), (Λ,Γ = 0.12, 0.04) was used, and in (D–F), (Λ,Γ = 0.01, 0.025) was used.(EPS)

S5 FigDependence of the simulation results on the cell division rule.(A, D) Comparison of phase diagrams for elimination success/failure between two different cell division rules (doubling and adder) for tissues with lower fluidity (A) and higher fluidity (D). The profile was not affected by the division rules. (B, E) The dependence of the average cell area ($1/\bar{\rho }$) on the interfacial contractility (*μ*Λ) for both division rules for tissues with lower fluidity (B) and higher fluidity (E): doubling (left) and adder (right). Note that the mechanical homeostatic cell density (*ρ*_1_) depends slightly on the division rules. (C) The difference between 1/ρ* and 1/*ρ*_1_ for two different cell division rules for tissues with lower fluidity. *ρ*_1_ was very close to ρ* independently of the division rules. (F) The difference between 1/ρ** and 1/*ρ*_2_ for two different cell division rules for tissues with higher fluidity. *ρ*_2_ was very close to ρ** independently of the division rules. In (A–C), (Λ,Γ = 0.12, 0.04) was used, and in (D–F), (Λ,Γ = 0.01, 0.025) was used.(EPS)
